# Association between keloids and cardiovascular disease in Black and Asian individuals

**DOI:** 10.1016/j.jdin.2025.06.003

**Published:** 2025-06-30

**Authors:** Karishma S. Shah, Michelle S. Min

**Affiliations:** Department of Dermatology, University of California Irvine School of Medicine, Irvine, California

**Keywords:** cardiovascular disease, epidemiology, hypertension, hypertrophic scars, keloids, systemic associations

*To the Editor:* Keloids are defined as scars that grow beyond their initial wound boundaries and are particularly prevalent among Black and Asian populations. Overactive fibroblasts lead to an increased ratio of type I collagen (COL1) to type 3 collagen (COL3) in keloids, but both are overexpressed in keloids compared to normal skin.^1^ Notably, COL1 and COL3 also provide structural support as part of myocardial extracellular matrices (ECM). While COL1 is found in most ECM, COL3 is also found in vasculature and hollow organs. Given collagen's role in the skin and cardiovascular system, we aimed to investigate the relationship between keloids and cardiovascular disease (CVD) in genetically at-risk populations.

Black and Asian patients, aged 18 years and up, with an International Classification of Diseases (ICD-10) code for hypertrophic scar/keloid (ICD-10, L91.0) from 2004 to 2024 were extracted from the TriNetX database, a global network comprising deidentified patient data. These cohorts were 1:1 matched by age, sex, race, ethnicity, tobacco use (Z72.0), alcohol use (F10), hyperlipidemia (E78.5), type 2 diabetes mellitus (E11), and overweight/obesity (E66) to controls without keloids. The primary outcome of interest was a CVD diagnosis, defined by 13 diseases affecting the myocardium/vasculature based on ICD codes (Table I). Analysis consisted of univariate logistic regression and chi-squared tests with *P*-value < .05 considered statistically significant.

**Table I:** Demographic data and differences in the presence of cardiovascular disease for Black and Asian patients with keloids and matched control patients

	Black patients			Asian patients		
	Keloids	Control	*P*	Keloids	Control	*P*
Patients after matching (*n*)	43,029	43,029		13,423	13,423	
Age at index (mean in years ± SD)∗	41.8 ± 16.8	41.8 ± 16.7	.75	40.7 ± 16.6	40.7 ± 16.6	.79
Female (*n*, %)∗	27,989 (65.1%)	27,922 (64.9%)	.63	8743 (65.1%)	8732 (65.1%)	.89
Hispanic† (*n*, %)∗	667 (1.6%)	666 (1.6%)	.98	121 (0.9%)	125 (0.9%)	.80
Cardiovascular risk factors∗						
Hyperlipidemia (*n*, %)	7094 (16.5%)	7088 (16.5%)	.97	1806 (13.5%)	1848 (13.8%)	.45
Type 2 diabetes mellitus (*n*, %)	6059 (14.1%)	6119 (14.2%)	.56	1178 (8.8%)	1195 (8.9%)	.71
Alcohol ue (*n*, %)	1215 (2.8%)	1218 (2.8%)	.95	118 (0.9%)	102 (0.8%)	.28
Tobacco use (*n*, %)	991 (2.3%)	1059 (2.5%)	.13	74 (0.6%)	74 (0.6%)	1.0
Overweight/obesity (*n*, %)	10,278 (23.9%)	10,215 (23.7%)	.61	914 (6.8%)	851 (6.3%)	.12
CVD, ICD-10 codes						
Essential primary hypertension, I10 (*n*, %)	15,928 (37.1%)	12,699 (29.5%)	<.001	2790 (20.8%)	2543 (19.0%)	<.001
Angina pectoris, I20 (*n*, %)	746 (1.7%)	417 (1.0%)	<.001	172 (1.3%)	128 (1.0%)	.01
Myocardial infarction, I21 (*n*, %)	953 (2.2%)	855 (2.0%)	.02	142 (1.1%)	161 (1.2%)	.27
Cardiomyopathy, I42 (*n*, %)	1547 (3.6%)	1210 (2.8%)	<.001	135 (1.0%)	145 (1.0%)	.55
Heart failure, I50 (*n*, %)	2917 (6.8%)	2668 (6.2%)	<.001	337 (2.5%)	322 (2.4%)	.55
Cerebral infarction, I63 (*n*, %)	1318 (3.1%)	944 (2.2%)	<.001	167 (1.5%)	158 (1.4%)	.62
Atherosclerosis, I70 (*n*, %)	1544 (3.6%)	1275 (3.0%)	<.001	264 (2.0%)	238 (1.8%)	.24
Aortic aneurysm and dissection, I71 (*n*, %)	389 (0.9%)	245 (0.6%)	<.001	115 (0.9%)	74 (0.6%)	.003
Nonaortic aneurysm, I72 (*n*, %)	251 (0.6%)	150 (0.4%)	<.001	57 (0.4%)	35 (0.3%)	.022
Arterial embolism and thrombosis, I74 (*n*, %)	210 (0.5%)	113 (0.3%)	<.001	28 (0.2%)	22 (0.2%)	.40
Atheroembolism, I75 (*n*, %)	27 (0.1%)	10 (0.02%)	.0052	10 (0.07%)	10 (0.07%)	1.00
Septic arterial embolism, I76 (*n*, %)	12 (0.03%)	10 (0.02%)	.67	10 (0.07%)	10 (0.07%)	1.00
Other arterial disease, I77 (*n*, %)	1090 (2.5%)	710 (1.7%)	<.001	248 (1.9%)	165 (1.2%)	<.001

*CVD*, Cardiovascular disease; *ICD-10*, International Classification of Disease; *SD*, standard deviation.

∗ Cohorts were 1:1 propensity score matched based upon these variables.

† Individuals who identified themselves as racially Black or Asian and ethnically Hispanic or Latino.

A total of 56,452 patients were included in our analysis (Table I). A majority of CVD diagnoses were significantly associated with keloids in Black patients (Fig 1). Atheroembolism had the highest significant odds ratio in Black patients with an odds ratio (OR) of 2.70 (95% confidence interval [CI]: 1.31-5.58). In Asian patients, non-aortic aneurysm had the highest significant odds ratio (OR: 1.63 (1.07-2.45). The most significant myocardial disease was angina pectoris in both Black (OR: 1.80, CI: 1.60-2.03) and Asian (OR: 1.35, CI: 1.07-1.70) patients.

**Fig 1:**
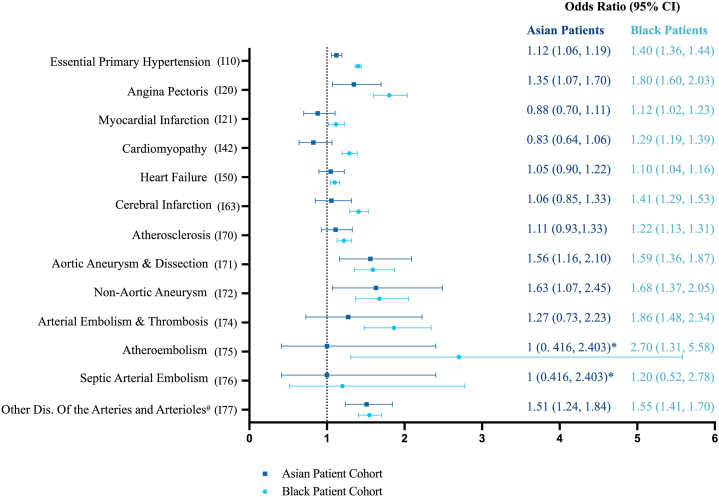
Keloids and cardiovascular disease. Odds ratios for various cardiovascular conditions in the setting of keloids in Black and Asian patients. ∗Denotes that there were <10 outcomes for analysis. #Dis. serves as an abbreviation for diseases.

Our study expands on emerging data suggesting that keloids are not an isolated dermatologic condition. Joint pain, uterine leiomyomas, and irritable bowel syndrome have been associated with keloids.^2^ Several independent studies have also linked hypertension with keloids,^3^ corroborating our findings. We now add other CVDs as potential associations. These findings suggest that aberrant collagen synthesis seen in keloids might not be limited to the dermis. Prior research has confirmed that disruption and subsequent accumulation of COL1/3 in the myocardial ECM play a crucial role in heart remodeling and the development of many CVDs, eventually resulting in heart failure.^4^ Additionally, COL3 influences vascular tensile strength and facilitates platelet adhesion.^5^ It is possible that hyperactive fibroblasts compromise vascular integrity and promote hypercoagulability. Interestingly, data suggest that statins have antifibrotic properties; thereby, statins could address both CVD and keloids,^6^ but future studies are warranted.

Study limitations include retrospective design and lack of differentiation between hypertrophic scars and keloids based on ICD code. Additionally, several confounders, such as major cardiovascular surgeries, could not be explored within this database.

Overall, awareness of the heightened risk of CVD in patients with keloids can lead to better understanding, counseling, and screening.

## Conflicts of interest

MSM is on the advisory boards of BMS and Horizon. She is an investigator for Amgen, BMS, BI, and Priovant.
